# Checkpoint Blockade Toxicity and Immune Homeostasis in the Gastrointestinal Tract

**DOI:** 10.3389/fimmu.2017.01547

**Published:** 2017-11-15

**Authors:** Michael Dougan

**Affiliations:** ^1^Division of Gastroenterology, Department of Medicine, Massachusetts General Hospital, Boston, MA, United States

**Keywords:** management, gastrointestinal diseases, cancer immunotherapy, immune-related adverse events, checkpoint blockade

## Abstract

Monoclonal antibodies targeting the regulatory immune “checkpoint” receptors CTLA-4, PD-1, and PD-L1 are now standard therapy for diverse malignancies including melanoma, lung cancer, and renal cell carcinoma. Although effective in many patients and able to induce cures in some, targeting these regulatory pathways has led to a new class of immune-related adverse events. In many respects, these immune toxicities resemble idiopathic autoimmune diseases, such as inflammatory bowel disease, autoimmune hepatitis, rheumatoid arthritis, and vitiligo. Understanding the pathogenesis of these immune toxicities will have implications not only for care of patients receiving checkpoint blockade but may also provide critical insights into autoimmune disease. The gastrointestinal (GI) mucosa is arguably the most complex barrier in the body, host to a diverse commensal microflora and constantly challenged by ingested foreign proteins both of which must be tolerated. At the same time, the GI mucosa must defend against pathogenic microorganisms while maintaining sufficient permeability to absorb nutrients. For these reasons, regulatory cells and receptors are likely to play a central role in maintaining the gut barrier and GI toxicities, such as colitis and hepatitis are indeed among the most common side effects of CTLA-4 blockade and to a lesser extent blockade of PD-1 and PD-L1. High-dose corticosteroids are typically effective for management of both checkpoint colitis and hepatitis, although a fraction of patients will require additional immune suppression such as infliximab. Prompt recognition and treatment of these toxicities is essential to prevent more serious complications.

## Introduction

Immune therapy has been a cornerstone of cancer therapy for decades, with tumor-targeting monoclonal antibodies, bone marrow transplant, and vaccines playing an important role in the treatment of multiple malignancies (1). Over the last decade, with the development of monoclonal antibodies that target the regulatory immune “checkpoint” receptors CTLA-4, PD-1, and PD-L1, immune therapy has now become standard therapy for diverse malignancies including melanoma, lung cancer, and renal cell carcinoma with the list of responsive tumors rapidly expanding (2–11). Each of these antibodies targets an IgV-domain containing immune receptor expressed by activated T cells (PD-1 and CTLA-4), or tumor or tumor-associated myeloid cells (PD-L1), that functions to inhibit antigen-dependent T cell responses. CTLA-4 is also highly expressed on regulatory T cells, and binding to these cells may be important to the function of these antibodies (2, 12–17).

Immune therapy is distinguished from other targeted therapies and chemotherapy not only through its mechanism of action but also through its effect on long-term survival (2, 11). By targeting the immune system, rather than the tumor itself, immune therapies can have beneficial effects in tumors arising from a wide range of organs, with responses appearing to correlate more with the degree of mutational load than with the specific mechanism of oncogenic transformation (18, 19). Because the immune system is itself adaptive, tumors may have more difficulty mutating to avoid an ongoing immune assault than to resist other targeted therapies or chemotherapy. Consequently, while patients who do not respond to immune therapy typically show survival similar to untreated patients, those who do respond have a higher likelihood of achieving long-term remissions than do patients treated with other modalities (2, 11). Thus, the median survival on immune therapy often underrepresents the impact of treatment, with the proportion of long-term survivors better reflecting the clinical importance of these drugs (2, 11).

Although most cancer patients do not respond to current immune therapies, a plethora of antibodies are in development that target other innate and adaptive immune regulatory receptors such TIM-3, LAG3, TIGIT, and CD47, as well as activating antibodies to T cell co-stimulatory proteins (e.g., OX40, CD137) (2). In addition, novel immune adjuvants, co-stimulatory small molecules, inhibitors of immunosuppressive pathways, cancer vaccines, and cellular therapies among others are being used alone or in combination with approved immune therapies to expand the range of patients who can benefit from these treatments (2, 20). Despite the immense promise of immune therapy for cancer, all of these treatments are to some extent limited by immune-related adverse events (irAEs), and effective management strategies for these toxicities will play an important role in enabling immune therapy to reach its full clinical potential (3–7, 9, 21, 22).

## Immune-Mediated Adverse Events

Toxicities related to the immune system are common in patients treated with checkpoint blockade and can affect every organ system of the body, with the severity and spectrum of organ system involvement dependent on the specific pathway targeted (Table 1) (3–7, 9, 21–28). CTLA-4 blockade by ipilimumab is considerably more toxic than any of the currently approved antibodies to PD-1 or PD-L1 (3–7, 9, 21–28). High-dose ipilimumab used as adjuvant therapy for melanoma, and combination therapy with anti-PD-1 antibodies increases the spectrum and severity of toxicity (3, 21, 29). While the expanded use of anti-PD-1 antibodies reduces the likelihood of toxicities in an individual patient, the growing number of patients under treatment has increased the prevalence of these toxicities. In addition, many exploratory immune therapies involve combination treatments that simultaneously target more than one regulatory pathway, which is likely to substantially increase the frequency of severe toxicities (2).

**Table 1:** Frequency of common toxicities associated with checkpoint blockade.

	Ipilimumab	αPD-1^a^	αPD-L1^b^	Ipilimumab + αPD-1
**Common toxicities of checkpoint blockade (all grades)**				

**Constitutional**
Fatigue	15.2–48	10.4–34.2	13.1–25	35.1–39
Asthenia	6.3–11	4.8–11.5	6.6	9
Pyrexia	6.8–15	4.2–10.4	6.6–8	18–20
**Dermatologic**
Pruritus	26–35.4	8.5–20	8–10	33.2–40
Rash	14.5–32.8	0.9–25.9	8	40.3–41
**Gastrointestinal (GI)**
Diarrhea	22.7–37	7.5–19.2	9.8–15	44.1–45
Nausea	8.6–24	5.7–16.5	6.6–17	21–25.9
Vomiting	7–11	2.6–16.4		13–15.3
Decreased appetite	9–12.5	1.9–10.9	8–8.2	12–17.9
Constipation	9	2–10.7		8–11
Colitis	8.2–11.6	0.9–3.6	2	18–23
Hepatitis	1.2–3.9	1.1–3.8	4	15.3–27
Increased lipase	14–17	0.6		13–18
**Musculoskeletal**
Arthalgia	5–9	2.8–14	6–10	10.5–11
**Endocrine**				
Hypothyroidism	1–15	4.8–11	5–8	15.3–17
Hyperthyroidism	2.3–4.2	3.2–7.8		
Hypophysitis	2–2.3	0.4–0.7		12–13
Adrenal insufficiency	0–2	0.4		5
**Pulmonary**				
Pneumonitis	0–1.8	0.4–5.8	4	9–11

**Common toxicities of checkpoint blockade (grades 3–5)**				

**Constitutional**
Fatigue	1–1.2	0.4–1.3	2	4.2–5
Asthenia	0–0.8	0.4–1		0
Pyrexia	0–0.3	0.6		0.6–3
**Dermatologic**
Pruritus	0–0.4	0	<1	1–1.9
Rash	0–1.9	0.5–3.6		4–5
**GI**
Diarrhea	3–11	1–3.9	<1	9.3–11
Nausea	0–2	0–0.8	<1	1–22
Vomiting	0–0.3	0.3–0.6		1–2.6
Decreased appetite	0–0.3	1		1–1.3
Constipation	0	0.4		1
Colitis	7–8.7	0.6–2.5		7.7–17
Hepatitis	0–0.4	0.6–1.8		6.1–11
Increased lipase	13	0.6		9
**Musculoskeletal**				
Arthalgia	0–0.8	0–0.4	<1	0–0.3
**Endocrine**
Hypothyroidism	0	0–0.4		0–0.3
Hyperthyroidism	0.4	0		
Hypophysitis	1.6–4	0.4–0.6		1–2
Adrenal insufficiency		0.4		
**Pulmonary**				
Pneumonitis	0.4–2	0.4–2.6		2

*All grades (top panel). Grade 3/4 toxicities only (bottom panel). Stated frequencies represent the range of reported toxicity in large clinical trials (3–7, 9, 21–28)*.

*^a^Nivolumab and pembrolizumab*.

*^b^Atezollzumab and durvatumab*.

The cellular and molecular mechanisms of effective antitumor responses enhanced by checkpoint blockade are beginning to be understood, as are the common resistance pathways (30–33). The mechanisms of toxicity are comparatively poorly worked out, and represent an important area for further research both for our basic understanding of immune regulation and to improve the quality of cancer care. The spectrum of toxicities observed with checkpoint blockade provides insights into the principal functions of these pathways in humans. Much like knockout experiments in preclinical model organisms (34), by inhibiting CTLA-4 and observing colitis, we learn that CTLA-4 plays an important role in the regulation of gut homeostasis. Similarly, when PD-1 blockade induces hepatitis or pneumonitis, we learn that the PD-1 pathway inhibits the responses of activated T cells in part to prevent autoimmune destruction of these critical organs.

By delving into the cellular and molecular details of the inflammatory responses induced by checkpoint blockade, we stand to gain substantial insights into the spontaneous autoimmune diseases that resemble these adverse events (e.g., ulcerative colitis or autoimmune hepatitis). Such an analysis has the potential to revealing critical steps in autoimmune disease onset, which could be used to develop novel early diagnostics, preventative measures, or novel treatment strategies.

Despite substantial overlap, that CTLA-4 and PD-1/PD-L1 blockade differ in the spectrum of organs involved by immune toxicities implies differences in the biology of these regulatory pathways and the factors that govern their use. Patients treated with combination CTLA-4 and PD-1 blockade develop more frequent and severe toxicities characteristic of both single agents, but do not appear to develop any irAEs that are unique to combination therapy (35). This provides further evidence that these mechanistically distinct regulatory pathways do not substitute for each other in regulating peripheral tolerance. Although mice and humans deficient in CTLA-4 develop a severe autoimmune syndrome that resembles some aspects of ipilimumab toxicity (34, 36–38), mice deficient in PD-1 or PD-L1 are relatively healthy, mirroring the differences in the severity of toxicities produced by targeting each pathway (39, 40). Intriguingly, mice treated with antibodies to CTLA-4, PD-1, and/or PD-L1 develop no significant toxicities, limiting their utility for understanding irAEs in patients, and emphasizing the importance of studying human samples if we are to develop a full pictures of irAEs (41, 42).

## Balancing irAEs with the Antitumor Response

A more nuanced view of immune-related toxicities also has the potential to have a significant impact on patient care. Presently, a substantial fraction of patients on checkpoint blockade develop grade 3/4 toxicities that lead to discontinuation of treatment, and in many cases necessitate initiation of high-potency systemic corticosteroids (3–7, 9, 21–28). Even in patients who develop grade 1 and grade 2 toxicities, therapeutic interruptions and treatment with local or systemic corticosteroids is fairly common. Although not formally demonstrated, it is likely that corticosteroids inhibit at least some elements of effective antitumor responses (43–45). Patients who develop severe toxicities that require high-dose corticosteroids for treatment do not appear to have a lower overall survival than those who do not develop these toxicities, and in fact trend toward increased survival; however, whether overall survival could be further improved by corticosteroid-sparing treatment strategies is presently unknown (43–45). Although some patients clearly do maintain productive antitumor responses despite systemic corticosteroids, corticosteroids may well limit ongoing productive antitumor immunity, preventing optimal responses to therapy. Indeed, the frequent use of systemic corticosteroids to treat toxicities may explain why combination therapy targeting CTLA-4 and PD-1 has a higher overall response rate in melanoma but does not appear to induce a substantial improvement in overall survival (3).

The goal of treatment for irAEs should be to preserve or replace organ function while minimizing the degree of systemic immune suppression, and if possible enabling ongoing antitumor therapy. For some organs such as the pituitary or thyroid, replacement is relatively straightforward, while inflammation targeting the heart or lungs requires some measure of immune suppression. The specific cellular and molecular immune mechanisms underlying toxicity are unlikely to precisely match those that cause tumor rejection. These differences may relate to particular immune cell subsets, or organ-specific homing signals (chemokines, integrins). For example, interferon-γ is likely a key player in tumor rejection, while evidence suggests that tumor necrosis factor (TNF)-α is of primary importance in the pathogenesis of ipilimumab-induced colitis (31, 46, 47). TNF-α may be entirely dispensable for the antitumor effect in most cases, making TNF-α a potential toxicity-specific target (47). By characterizing the differences between the antitumor response and the inflammatory toxicities, we should be able to identify new therapeutic targets that can preferentially inhibit toxicities while preserving the response to the tumor. Doubtlessly, almost any immune suppression will have less of a general effect than systemic corticosteroids.

## Gastrointestinal (GI) Toxicities of Checkpoint Blockade

To some extent, the organs targeted in irAEs make conceptual sense. The most common toxicities occur at barriers, where the immune system interacts with the outside world, including the skin, GI mucosa and liver, and lungs (Table 1). Endocrine organs with tissue-specific protein production such as the pituitary, thyroid, and pancreas are also targeted in a subset of patients, and similar toxicities are well described in many genetic defects of immune regulation (36–38, 48, 49). The GI toxicities of checkpoint blockade represent an excellent model for developing a framework to understand checkpoint blockade toxicities more generally.

The GI mucosa is arguably the most complex barrier in the body, host to a diverse commensal microflora and constantly challenged by ingested foreign proteins both of which must be tolerated (50, 51). At the same time the GI mucosa is charged with defending against pathogenic microorganisms and enabling sufficient permeability to absorb nutrients (50, 51). That this barrier requires effective immune control is expected, with disruption of the normal mechanisms of immune regulation likely to interfere with the subtle distinction between tolerated normal microflora or food, and dangerous invading pathogens.

**Figure 1:**
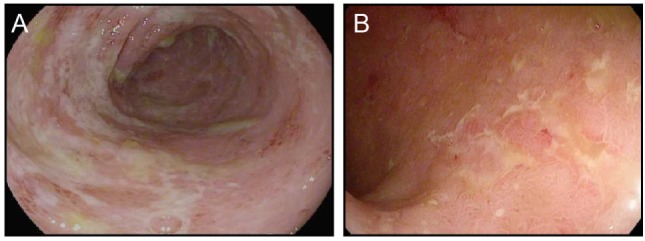
Ipilimumab colitis endoscopically resembles ulcerative colitis. **(A,B)** High resolution photograph of the colon of a patient with ipilimumab-associated colitis **(A)** or with ulcerative colitis **(B)**.

**Figure 2:**
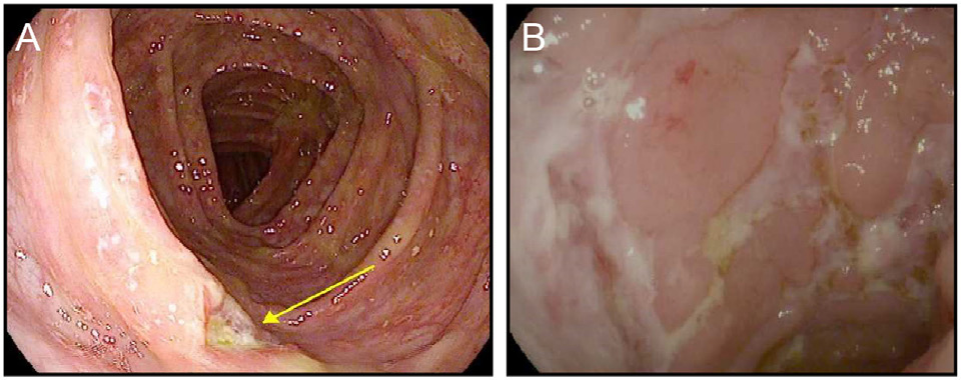
Ipilimumab colitis with isolated deep ulceration. **(A,B)** high resolution photograph of the colon of a patient with ipilimumab-associated colitis showing an isolated deep ulceration typical of Crohn’s disease **(A)** or similar ulcers found in a patient with “sporadic” Crohn’s disease **(B)**.

CTLA-4 appears to play a more central role in gut homeostasis than do either PD-1 or PD-L1. Mild colitis is common in patients on ipilimumab, with diarrhea affecting nearly half of the patients on high dose or combination therapy (3–5, 29). Severe colonic inflammation (colitis) is less frequent, but still occurs in a substantial fraction of patients, and can be life-threatening (3–5, 29). Ipilimumab-induced (checkpoint) colitis most closely resembles pan-colonic ulcerative colitis, a subset of “sporadic” inflammatory bowel disease (IBD), with continuous inflammation from the anus to the cecum (Figure 1). This inflammation is characterized by edema, erythema, and friability, with diffuse shallow ulcerations occurring in the most severe cases. Segmental disease with deep ulcerations, as found in Crohn’s disease, the other major subset of sporadic IBD, is much less common (Figure 2) (52). Fistulas and strictures, also characteristic of Crohn’s disease, do not appear to occur. Although most patients develop colitis, approximately a quarter of patients with colonic involvement also have diffuse inflammation in the small intestines (enteritis) (46). This can lead to disproportionate diarrhea, and is a distinction from sporadic IBD, where ulcerative colitis is exclusively confined to the colon, and where, in Crohn’s disease, continuous inflammation from the small bowel through the colon is extremely rare. In patients with diffuse enteritis (or enterocolitis), the clinical presentation more closely resembles a severe infectious enterocolitis. Histopathologically, ipilimumab-induced colitis (as well as enteritis) has a high proportion of lymphocytes and increased numbers of apoptotic epithelial cells; granulomas are rare, as are chronic changes to the epithelial architecture (52). This contrasts to patients with clinically active (flaring) IBD, who exhibit frequent acute neutrophilic infiltrates and have a high prevalence of chronic changes to the epithelial structure. Understanding the mechanistic basis for the distinctions between sporadic IBD and checkpoint colitis may provide important insights into the pathophysiology of IBD.

**Figure 3:**
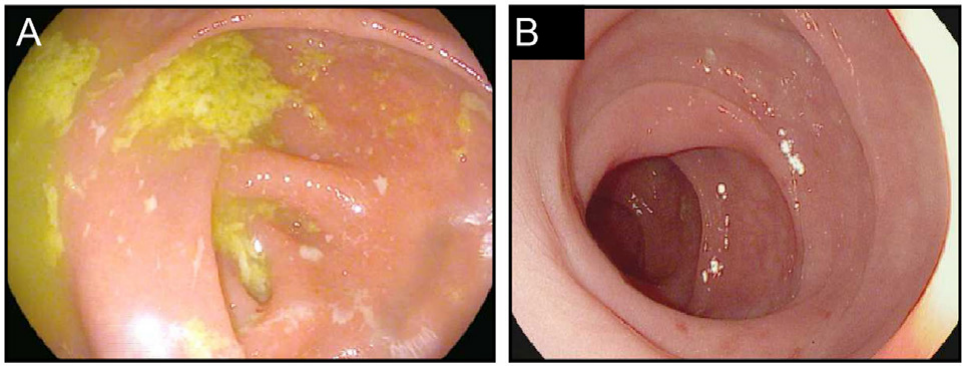
Colitis associated with PD-1 blockade. **(A,B)** High resolution photograph of the colon from a patient with moderate to severe colitis associated with PD-1 blockade **(A)** or a patient with biopsy confirmed PD-1-blockade-associated microscopic colitis **(B)**.

PD-1/PD-L1 blockade induces small intestinal and colonic inflammation that is clinically distinct from the colitis induced by ipilimumab. A relatively high frequency of patients treated with PD-1/PD-L1 blockade develop low-grade diarrhea, but this rarely progresses to severe colitis (3, 7, 9, 26, 28, 53) (Figure 3). In many cases, pathophysiologically, this low-grade diarrhea represents either isolated enteritis, or colitis that appears normal on endoscopy, and on biopsy resembles lymphocytic (or microscopic) colitis (Figure 3). Although severe colonic inflammation is much less common with PD-1/PD-L1 blockade than it is with ipilimumab, inflammation of the lungs (pneumonitis) is more strongly associated with inhibition of the PD-1/PD-L1 pathway, emphasizing the different regulatory roles of these receptors within distinct tissues (Table 1).

**Figure 4:**
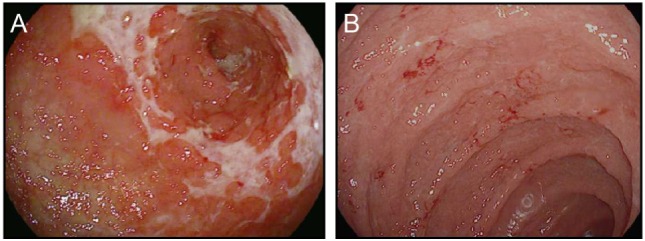
Upper gastrointestinal manifestations of checkpoint blockade. **(A,B)** High resolution photograph of the stomach **(A)** or the duodenum **(B)** from a patient with severe checkpoint blockade-associated gastritis **(A)** or enteritis **(B)**.

In addition to the relatively common enterocolitis and inflammation of the liver (hepatitis), isolated cases of symptomatic and asymptomatic pancreatitis, gastritis, and Celiac disease have been reported with checkpoint blockade (54–56) (Figure 4). Intriguingly, food allergies have not been observed to arise or worsen during treatment with either class of checkpoint blockade. This finding suggests that neither CTLA-4 nor PD-1/PD-L1 plays a substantial role in the regulation of oral tolerance to food antigens in humans (55).

## Management of Checkpoint Blockade Colitis

Prompt diagnosis is the most critical aspect of management for checkpoint blockade colitis. When recognized and treated early, severe complications are rare. With appropriate clinical suspicion, most treatment algorithms recommend diagnosis of grade 1 and 2 toxicities based on history after infectious colitis has been excluded (57, 58). Statistics on the relative risk of infectious colitis in this population are not presently available. Testing for *C. difficile* colitis using an antigen-based assay is a reasonable addition to standard stool cultures, given the exposure to a health-care setting. PCR based tests for *C. Difficile* are too sensitive, and may lead to misdiagnosis of patients who are colonized with *C. difficile* but who do not have active infection. Testing for parasitic infections should be based on physician-determined pre-test risk, reflecting prevalence of these infections in the surrounding population as well as travel history.

Colitis most frequently occurs between the second and third ipilimumab dose, though it can occur at any point during treatment. Colitis from PD-1/PD-L1 is less predictable, and while early occurrence can happen, many patients present months or even years into therapy (58). Presentation of enterocolitis is typically increased frequency of loose/watery stools, urgency, and cramping. Abdominal pain is usually modest, and bloody stools and nocturnal symptoms are rare. In patients with involvement of the upper GI tract (stomach, small intestines), nausea, vomiting, early satiety, and bloating can also be prominent symptoms.

Cross-sectional imaging (generally computed tomography) is unnecessary in grade 1 and 2 disease, but can be used to suggest the diagnosis in most patients with symptoms of more severe grade 3/4 colitis, although there are no characteristic imaging findings of checkpoint colitis (59). Cross-sectional imaging should be strongly considered in any patient with fever or severe pain to exclude perforation or abscess. Endoscopic evaluation with biopsies is the gold standard for diagnosis and has the advantage of providing information on the mucosal severity and extent of disease, which can be used to guide decisions about continuation of immune therapy. Biopsy also has the advantage of distinguishing checkpoint colitis from less common causes of diarrhea in these patients, such as infection with cytomegalovirus (CMV). The decision to confirm the diagnosis endoscopically prior to treatment should be based, in part, on the availability of these diagnostics, as well as clinical suspicion for alternative etiologies, and the likelihood that treatment decisions will be made based on the severity of endoscopic involvement. The type of endoscopy performed should be tailored to the symptoms of the patient. In general, a flexible sigmoidoscopy is sufficient to provide diagnostic information in approximately 95% of patients with suspected ipilimumab colitis, although whether this is true for PD-1/PD-L1 blockade colitis is not yet clear, particularly given the occurrence of isolated enteritis in these patients (52).

Once diagnosed, therapy is dependent on grade. Grade 1 and most grade 2 enterocolitis can be managed symptomatically with loperamide or diphenoxylate/atropine, and potentially with treatment delay (58). Persistent grade 2 enterocolitis, and nearly all grade 3/4 enterocolitis will typically require management with systemic corticosteroids (44, 57, 58). In patients with microscopic colitis, regardless of the severity of the diarrhea, local treatment with budesonide is a reasonable alternative. For grade 3/4 enterocolitis, discontinuation of treatment is recommended in addition to initiation of systemic corticosteroids (58); the distinction between intravenous (IV) and oral corticosteroids should be made based on the overall stability of the patient, but a single dose of IV corticosteroids delivered in an infusion clinic prior to starting oral corticosteroids may help patients avoid hospitalization. Corticosteroids should generally be tapered over a period of 1–2 months depending on the severity of the disease (58).

Although most patients respond to corticosteroids, corticosteroid refractory patients, and patients with recurrence during corticosteroid taper make up about a third of colitis cases, and may require alternative treatments (46, 58). The best-studied alternative to corticosteroids is the anti-TNF-α antibody infliximab, which has been used in a few small case series and several clinical trials, and typically resolves inflammation within 1–3 doses (46, 58). Symptom resolution with infliximab is usually within days to a few weeks. Prior to initiation of infliximab, diagnosis should be confirmed endoscopically, and patients should be confirmed to be uninfected by Hepatitis B and *Mycobacterium tuberculosis*. Infliximab is used to treat patients with ulcerative colitis as well as Crohn’s disease, and is active in a number of rheumatologic syndromes. The fact that infliximab leads to rapid improvement in the majority of treated patients strongly suggests that TNF-α-mediated inflammation plays a central role in checkpoint enterocolitis. A single retrospective study found a slight increase in melanoma risk in patients treated with anti-TNF-α therapy for IBD, although no such relationship was found in larger series that have pooled patients receiving anti-TNF-α therapy for a variety of indications (60, 61). Although this provides some circumstantial evidence that infliximab may not be optimal for patients receiving immunotherapy for melanoma, TNF-α signaling has not been implicated in detailed assessments of correlates of effective antitumor responses to checkpoint blockade, nor has downstream TNF-α signaling been implicated in resistance to therapy (30–33). Furthermore, a retrospective analysis of patients receiving anti-TNF-α treatment for checkpoint enterocolitis showed a trend toward improved outcomes (47). Taken together, these preliminary findings provide a strong rationale for the safety of TNF-α blockade in patients receiving immunotherapy, suggesting that TNF-α does not play an important role in effective antitumor responses induced by checkpoint blockade, and may even indicate a therapeutic benefit.

Infrequently, patients with enterocolitis from ipilimumab or PD-1/PD-L1 blockade develop disease that is refractory to both corticosteroids and infliximab. Although the predictors of unresponsiveness to corticosteroids and infliximab have not been rigorously defined, some of these patients have underlying IBD. Immunotherapy can be used safely in patients with quiescent IBD, though when these patients develop colitis it is often difficult to manage (62); treatment of patients with active IBD with immunotherapy should be avoided. In any patient who fails corticosteroids and infliximab regardless of underlying predispositions, colonic infections such as CMV should be excluded endoscopically. If persistent inflammation is confirmed, and infections are excluded, other treatments derived from the experience with IBD can be considered.

The α4β7 gut homing integrin inhibitor, vedolizumab, has been reported to be effective in a small number of patients, although the time to response was quite slow (58, 63). Ustekinumab, a monoclonal antibody against IL-23p40, is another reasonable alternative, though this has not been directly studied as a treatment for colitis from checkpoint blockade (64). The Janus kinase inhibitor tofacitinib is effective in Rheumatoid arthritis and has promising efficacy in ulcerative colitis (65). Mycophenolate mofetil, and tacrolimus are other reasonable considerations, and for less severe disease, both azathioprine and low dose methotrexate should be considered. Switching to alternative anti-TNF-α therapies such as golimumab, adalimumab, and certolizumab pegol may be effective. Fecal microbiota transplant is an investigational approach that may have promise as well, though presently very little is known about the microbial features of this syndrome (66). As a treatment of last resort, total parental nutrition may be effective, and colectomy can be used for patients whose disease is isolated to the colon.

## Conclusion

Effective management of irAEs will require a detailed understanding of the molecular and cellular pathways involved in the toxicity. This mechanistic understanding will be particularly important in the setting of combination immunotherapy, which is likely to be the forefront of treatment in the future. Research efforts should focus on identifying distinct cells types, critical signaling cascades, and/or cytokines/chemokines involves in propagating or initiating toxicity. Through these mechanistic efforts, we can hope to identify novel diagnostics, either for identifying high-risk patients or for detecting toxicities before they begin to alter organ function. In addition, a better understanding of mechanism should enable identification of therapeutic strategies that could shut down organ-specific inflammation, while preserving the critical elements of the antitumor response. Infliximab may well be such a therapeutic approach for colitis, although this remains to be more rigorously established. Blockade of gut homing integrins represents another attractive therapeutic strategy. The antibody vedolizumab, which binds to the gut homing integrin α4β7 is effective in IBD, and may be able to prevent entry of immune cells into the colon in checkpoint colitis without altering trafficking into the tumor. Doubtlessly, similar strategies are yet to be identified for colitis, as well as the many other organ-specific inflammatory diseases induced by CTLA-4 and PD-1/PD-L1 targeted therapies. The effort to uncover these mechanisms may yield additional valuable insights into the etiology and pathogenesis of sporadic autoimmune diseases. By studying patients whose disease has a known cause, time of onset, and duration, we are likely to be able to distinguish primary events from secondary consequences. From there, we can begin to unravel some of the complexity of autoimmunity with implications for management of these diseases and for our basic understanding of immune regulation.

## Author Contributions

The author conceived of and wrote the manuscript.

## Conflict of Interest Statement

The author declares that the research was conducted in the absence of any commercial or financial relationships that could be construed as a potential conflict of interest.

## References

[1] Dougan M Dranoff G. Immune therapy for cancer. Annu Rev Immunol (2009) 27:83–117. 10.1146/annurev.immunol.021908.132544 19007331

[2] Baumeister SH Freeman GJ Dranoff G Sharpe AH. Coinhibitory pathways in immunotherapy for cancer. Annu Rev Immunol (2016) 34:539–73. 10.1146/annurev-immunol-032414-112049 26927206

[3] Larkin J Chiarion-Sileni V Gonzalez R Grob JJ Cowey CL Lao CD Combined nivolumab and ipilimumab or monotherapy in untreated melanoma. N Engl J Med (2015) 373:23–34. 10.1056/NEJMoa1504030 26027431 PMC5698905

[4] Hodi FS O’Day SJ McDermott DF Weber RW Sosman JA Haanen JB Improved survival with ipilimumab in patients with metastatic melanoma. N Engl J Med (2010) 363:711–23. 10.1056/NEJMoa1003466 20525992 PMC3549297

[5] Robert C Thomas L Bondarenko I O’Day S Weber J Garbe C Ipilimumab plus dacarbazine for previously untreated metastatic melanoma. N Engl J Med (2011) 364:2517–26. 10.1056/NEJMoa1104621 21639810

[6] Postow MA Chesney J Pavlick AC Robert C Grossmann K McDermott D Nivolumab and ipilimumab versus ipilimumab in untreated melanoma. N Engl J Med (2015) 372:2006–17. 10.1056/NEJMoa1414428 25891304 PMC5744258

[7] Robert C Long GV Brady B Dutriaux C Maio M Mortier L Nivolumab in previously untreated melanoma without BRAF mutation. N Engl J Med (2015) 372:320–30. 10.1056/NEJMoa1412082 25399552

[8] Le DT Uram JN Wang H Bartlett BR Kemberling H Eyring AD PD-1 blockade in tumors with mismatch-repair deficiency. N Engl J Med (2015) 372:2509–20. 10.1056/NEJMoa1500596 26028255 PMC4481136

[9] Garon EB Rizvi NA Hui R Leighl N Balmanoukian AS Eder JP Pembrolizumab for the treatment of non-small-cell lung cancer. N Engl J Med (2015) 372:2018–28. 10.1056/NEJMoa1501824 25891174

[10] Ansell SM Lesokhin AM Borrello I Halwani A Scott EC Gutierrez M PD-1 blockade with nivolumab in relapsed or refractory Hodgkin’s lymphoma. N Engl J Med (2015) 372:311–9. 10.1056/NEJMoa1411087 25482239 PMC4348009

[11] Pardoll DM The blockade of immune checkpoints in cancer immunotherapy. Nat Rev Cancer (2012) 12(4):252–64. 10.1038/nrc3239 22437870 PMC4856023

[12] Simpson TR Li F Montalvo-Ortiz W Sepulveda MA Bergerhoff K Arce F Fc-dependent depletion of tumor-infiltrating regulatory T cells co-defines the efficacy of anti-CTLA-4 therapy against melanoma. J Exp Med (2013) 210:1695–710. 10.1038/ni.1774 23897981 PMC3754863

[13] Selby MJ Engelhardt JJ Quigley M Henning KA Chen T Srinivasan M Anti-CTLA-4 antibodies of IgG2a isotype enhance antitumor activity through reduction of intratumoral regulatory T cells. Cancer Immunol Res (2013) 1:32–42. 10.1158/2326-6066.CIR-13-0013 24777248

[14] Bulliard Y Jolicoeur R Windman M Rue SM Ettenberg S Knee DA Activating Fc γ receptors contribute to the antitumor activities of immunoregulatory receptor-targeting antibodies. J Exp Med (2013) 210:1685–93. 10.4049/jimmunol.179.11.7365 23897982 PMC3754864

[15] Romano E Kusio-Kobialka M Foukas PG Baumgaertner P Meyer C Ballabeni P Ipilimumab-dependent cell-mediated cytotoxicity of regulatory T cells ex vivo by nonclassical monocytes in melanoma patients. Proc Natl Acad Sci USA (2015) 112:6140–5. 10.1073/pnas.0712237105 25918390 PMC4434760

[16] Arlauckas SP Garris CS Kohler RH Kitaoka M Cuccarese MF Yang KS In vivo imaging reveals a tumor-associated macrophage-mediated resistance pathway in anti-PD-1 therapy. Sci Transl Med (2017) 9(389):ii:eaal3604. 10.1126/scitranslmed.aal3604 28490665 PMC5734617

[17] Sharpe AH Freeman GJ The B7–CD28 superfamily. Nat Rev Immunol (2002) 2:116–26. 10.1038/35069118 11910893

[18] Rizvi NA Hellmann MD Snyder A Kvistborg P Makarov V Havel JJ Cancer immunology. mutational landscape determines sensitivity to PD-1 blockade in non-small cell lung cancer. Science (2015) 348:124–8. 10.1126/science.aaa1348 25765070 PMC4993154

[19] Schumacher TN Schreiber RD. Neoantigens in cancer immunotherapy. Science (2015) 348:69–74. 10.1126/science.aaa4971 25838375

[20] Ott PA Hu Z Keskin DB Shukla SA Sun J Bozym DJ An immunogenic personal neoantigen vaccine for patients with melanoma. Nature (2017) 547:217–21. 10.1038/nature22991 28678778 PMC5577644

[21] Hodi FS Chesney J Pavlick AC Robert C Grossmann KF McDermott DF Combined nivolumab and ipilimumab versus ipilimumab alone in patients with advanced melanoma: 2-year overall survival outcomes in a multicentre, randomised, controlled, phase 2 trial. Lancet Oncol (2016) 17:1558–68. 10.1016/S1470-2045(16)30366-7 27622997 PMC5630525

[22] Rittmeyer A Barlesi F Waterkamp D Park K Ciardiello F von Pawel J Atezolizumab versus docetaxel in patients with previously treated non-small-cell lung cancer (OAK): a phase 3, open-label, multicentre randomised controlled trial. Lancet (2017) 389:255–65. 10.1016/S0140-6736(16)32517-X 27979383 PMC6886121

[23] Peters S Gettinger S Johnson ML Jänne PA Garassino MC Christoph D Phase II trial of atezolizumab as first-line or subsequent therapy for patients with programmed death-ligand 1-selected advanced non-small-cell lung cancer (BIRCH). J Clin Oncol (2017) 35(24):2781–9. 10.1200/JCO.2016.71.9476 28609226 PMC5562171

[24] Massard C Gordon MS Sharma S Rafii S Wainberg ZA Luke J Safety and efficacy of durvalumab (MEDI4736), an anti–programmed cell death ligand-1 immune checkpoint inhibitor, in patients with advanced urothelial bladder cancer. J Clin Oncol (2016) 34:3119–25. 10.1200/JCO.2016.67.9761 27269937 PMC5569690

[25] Larkin J Lao CD Urba WJ McDermott DF Horak C Jiang J Efficacy and safety of nivolumab in patients with BRAFV600 mutant and BRAF wild-type advanced melanoma. JAMA Oncol (2015) 1:433 10.1001/jamaoncol.2015.1184 26181250

[26] Bellmunt J de Wit R Vaughn DJ Fradet Y Lee J-L Fong L Pembrolizumab as second-line therapy for advanced urothelial carcinoma. N Engl J Med (2017) 376:1015–26. 10.1056/NEJMoa1613683 28212060 PMC5635424

[27] Schachter J Ribas A Long GV Arance A Grob JJ Mortier L Pembrolizumab versus ipilimumab for advanced melanoma: final overall survival results of a multicentre, randomised, open-label phase 3 study (KEYNOTE-006). Lancet (2017) 390(10105):1853–62. 10.1016/S0140-6736(17)31601-X 28822576

[28] Reck M Rodríguez-Abreu D Robinson AG Hui R Csőszi T Fülöp A Pembrolizumab versus chemotherapy for PD-L1-positive non-small-cell lung cancer. N Engl J Med (2016) 375:1823–33. 10.1056/NEJMoa1606774 27718847

[29] Eggermont AMM Chiarion-Sileni V Grob JJ Dummer R Wolchok JD Schmidt H Prolonged survival in stage III melanoma with ipilimumab adjuvant therapy. N Engl J Med (2016) 375:1845–55. 10.1056/NEJMoa1611299 27717298 PMC5648545

[30] Wei SC Levine JH Cogdill AP Zhao Y Anang N-AAS Andrews MC Distinct cellular mechanisms underlie anti-CTLA-4 and anti-PD-1 checkpoint blockade. Cell (2017) 170:1120.e–33.e. 10.1016/j.cell.2017.07.024 28803728 PMC5591072

[31] Zaretsky JM Garcia-Diaz A Shin DS Escuin-Ordinas H Hugo W Hu-Lieskovan S Mutations associated with acquired resistance to PD-1 blockade in melanoma. N Engl J Med (2016) 375:819–29. 10.1056/NEJMoa1604958 27433843 PMC5007206

[32] Hugo W Zaretsky JM Sun L Song C Moreno BH Hu-Lieskovan S Genomic and transcriptomic features of response to anti-PD-1 therapy in metastatic melanoma. Cell (2016) 165:35–44. 10.1016/j.cell.2016.02.065 26997480 PMC4808437

[33] Rooney MS Shukla SA Wu CJ Getz G Hacohen N. Molecular and genetic properties of tumors associated with local immune cytolytic activity. Cell (2015) 160:48–61. 10.1016/j.cell.2014.12.033 25594174 PMC4856474

[34] Tivol EA Borriello F Schweitzer AN Lynch WP Bluestone JA Sharpe AH. Loss of CTLA-4 leads to massive lymphoproliferation and fatal multiorgan tissue destruction, revealing a critical negative regulatory role of CTLA-4. Immunity (1995) 3:541–7. 10.1016/1074-7613(95)90125-6 7584144

[35] Sznol M Ferrucci PF Hogg D Atkins MB Wolter P Guidoboni M Pooled analysis safety profile of nivolumab and ipilimumab combination therapy in patients with advanced melanoma. J Clin Oncol (2017). 10.1200/JCO.2016.72.1167 28915085

[36] Kuehn HS Ouyang W Lo B Deenick EK Niemela JE Avery DT Immune dysregulation in human subjects with heterozygous germline mutations in CTLA4. Science (2014) 345:1623–7. 10.1126/science.1255904 25213377 PMC4371526

[37] Zeissig S Petersen B-S Tomczak M Melum E Huc-Claustre E Dougan SK Early-onset Crohn’s disease and autoimmunity associated with a variant in CTLA-4. Gut (2015) 64:1889–97. 10.1136/gutjnl-2014-308541 25367873 PMC4512923

[38] Schubert D Bode C Kenefeck R Hou TZ Wing JB Kennedy A Autosomal dominant immune dysregulation syndrome in humans with CTLA4 mutations. Nat Med (2014) 20:1410–6. 10.1038/sj.leu.2403202 25329329 PMC4668597

[39] Nishimura H Nose M Hiai H Minato N Honjo T. Development of lupus-like autoimmune diseases by disruption of the PD-1 gene encoding an ITIM motif-carrying immunoreceptor. Immunity (1999) 11:141–51. 10.1016/S1074-7613(00)80089-8 10485649

[40] Latchman YE Liang SC Wu Y Chernova T Sobel RA Klemm M PD-L1-deficient mice show that PD-L1 on T cells, antigen-presenting cells, and host tissues negatively regulates T cells. Proc Natl Acad Sci USA (2004) 101:10691–6. 10.1073/pnas.0307252101 15249675 PMC489996

[41] van Elsas A Hurwitz AA Allison JP. Combination immunotherapy of B16 melanoma using anti-cytotoxic T lymphocyte-associated antigen 4 (CTLA-4) and granulocyte/macrophage colony-stimulating factor (GM-CSF)-producing vaccines induces rejection of subcutaneous and metastatic tumors accompanied by autoimmune depigmentation. J Exp Med (1999) 190:355–66. 10430624 10.1084/jem.190.3.355 PMC2195583

[42] Curran MA Montalvo W Yagita H Allison JP PD-1 and CTLA-4 combination blockade expands infiltrating T cells and reduces regulatory T and myeloid cells within B16 melanoma tumors. Proc Natl Acad Sci USA (2010) 107:4275–80. 10.4049/jimmunol.175.3.1586 20160101 PMC2840093

[43] Schadendorf D Wolchok JD Hodi FS Chiarion-Sileni V Gonzalez R Rutkowski P Efficacy and safety outcomes in patients with advanced melanoma who discontinued treatment with nivolumab and ipilimumab because of adverse events: a pooled analysis of randomized phase II and III trials. J Clin Oncol (2017). 10.1200/JCO.2017.73.2289 PMC5791828 28841387

[44] Horvat TZ Adel NG Dang T-O Momtaz P Postow MA Callahan MK Immune-related adverse events, need for systemic immunosuppression, and effects on survival and time to treatment failure in patients with melanoma treated with ipilimumab at memorial sloan kettering cancer center. J Clin Oncol (2015) 33:3193–8. 10.1200/JCO.2015.60.8448 26282644 PMC5087335

[45] Downey SG Klapper JA Smith FO Yang JC Sherry RM Royal RE Prognostic factors related to clinical response in patients with metastatic melanoma treated by CTL-associated antigen-4 blockade. Clin Cancer Res (2007) 13:6681–8. 10.1158/1078-0432.CCR-07-0187 17982122 PMC2147083

[46] Beck KE Blansfield JA Tran KQ Feldman AL Hughes MS Royal RE Enterocolitis in patients with cancer after antibody blockade of cytotoxic T-lymphocyte-associated antigen 4. J Clin Oncol (2006) 24:2283–9. 10.1200/JCO.2005.04.5716 16710025 PMC2140223

[47] Arriola E Wheater M Karydis I Thomas G Ottensmeier C Infliximab for IPILIMUMAB-related colitis – letter. Clin Cancer Res (2015) 21:5642–3. 10.1158/1078-0432.CCR-15-2471 26672088

[48] Cutolo M. Autoimmune polyendocrine syndromes. Autoimmun Rev (2014) 13:85–9. 10.1016/j.autrev.2013.07.006 24055063

[49] Cheng MH Anderson MS. Monogenic autoimmunity. Annu Rev Immunol (2012) 30:393–427. 10.1146/annurev-immunol-020711-074953 22224765 PMC6249029

[50] Kurashima Y Kiyono H. Mucosal ecological network of epithelium and immune cells for gut homeostasis and tissue healing. Annu Rev Immunol (2017) 35:119–47. 10.1146/annurev-immunol-051116-052424 28125357

[51] Strober W Fuss IJ Blumberg RS. The immunology of mucosal models of inflammation. Annu Rev Immunol (2002) 20:495–549. 10.1146/annurev.immunol.20.100301.064816 11861611

[52] Marthey L Mateus C Mussini C Nachury M Nancey S Grange F Cancer immunotherapy with anti-CTLA-4 monoclonal antibodies induces an inflammatory bowel disease. J Crohns Colitis (2016) 10:395–401. 10.1093/ecco-jcc/jjv227 26783344 PMC4946758

[53] Brahmer J Reckamp KL Baas P Crinò L Eberhardt WEE Poddubskaya E Nivolumab versus docetaxel in advanced squamous-cell non-small-cell lung cancer. N Engl J Med (2015) 373:123–35. 10.1056/NEJMoa1504627 26028407 PMC4681400

[54] Gentile NM D’Souza A Fujii LL Wu TT Murray JA. Association between ipilimumab and celiac disease. Mayo Clin Proc (2013) 88:414–7. 10.1016/j.mayocp.2013.01.015 23541015

[55] Abdel-Wahab N Shah M Suarez-Almazor ME. Adverse events associated with immune checkpoint blockade in patients with cancer: a systematic review of case reports. PLoS One (2016) 11:e0160221. 10.1371/journal.pone.0160221.s004 27472273 PMC4966895

[56] Kobayashi M Yamaguchi O Nagata K Nonaka K Ryozawa S Acute hemorrhagic gastritis after nivolumab treatment. Gastrointes Endosc (2017) 86(5):915–6. 10.1016/j.gie.2017.04.033 28478027

[57] Friedman CF Proverbs-Singh TA Postow MA Treatment of the immune-related adverse effects of immune checkpoint inhibitors: a review. JAMA Oncol (2016) 2:1346–53. 10.1001/jamaoncol.2016.1051 27367787

[58] Haanen JBAG Carbonnel F Robert C Kerr KM Peters S Larkin J Management of toxicities from immunotherapy: ESMO clinical practice guidelines for diagnosis, treatment and follow-up†. Ann Oncol (2017) 28:iv119–42. 10.1093/annonc/mdx225 28881921

[59] Kim KW Ramaiya NH Krajewski KM Shinagare AB Howard SA Jagannathan JP Ipilimumab-associated colitis: CT findings. AJR Am J Roentgenol (2013) 200:W468–74. 10.2214/AJR.12.9751 23718569

[60] Chen Y Sun J Yang Y Huang Y Liu G Malignancy risk of anti-tumor necrosis factor alpha blockers: an overview of systematic reviews and meta-analyses. Clin Rheumatol (2015) 35:1–18. 10.3109/09546634.2011.652064 26573205

[61] Maneiro JR Souto A Gomez-Reino JJ. Risks of malignancies related to tofacitinib and biological drugs in rheumatoid arthritis: systematic review, meta-analysis, and network meta-analysis. Semin Arthritis Rheum (2017) 47(2):149–56. 10.1016/j.semarthrit.2017.02.007 28284845

[62] Johnson DB Sullivan RJ Ott PA Carlino MS Khushalani NI Ye F Ipilimumab therapy in patients with advanced melanoma and preexisting autoimmune disorders. JAMA Oncol (2016) 2(2):234–40. 10.1001/jamaoncol.2015.4368 26633184

[63] Bergqvist V Hertervig E Gedeon P Kopljar M Griph H Kinhult S Vedolizumab treatment for immune checkpoint inhibitor-induced enterocolitis. Cancer Immunol Immunother (2017) 66:581–92. 10.1007/s00262-017-1962-6 28204866 PMC5406433

[64] Feagan BG Sandborn WJ Gasink C Jacobstein D Lang Y Friedman JR Ustekinumab as induction and maintenance therapy for Crohn’s disease. N Engl J Med (2016) 375(20):1946–60. 10.1056/NEJMoa1602773 27959607

[65] Sandborn WJ Su C Sands BE D’Haens GR Vermeire S Schreiber S Tofacitinib as induction and maintenance therapy for ulcerative colitis. N Engl J Med (2017) 376(18):1723–36. 10.1056/NEJMoa1606910 28467869

[66] Dubin K Callahan MK Ren B Khanin R Viale A Ling L Intestinal microbiome analyses identify melanoma patients at risk for checkpoint-blockade-induced colitis. Nat Commun (2016) 7:10391. 10.1038/ncomms10391 26837003 PMC4740747

